# Supplementary material for: Multimorbidity Management: A Scoping Review of Interventions and Health Outcomes
Source: Int J Environ Res Public Health. 2025 May 13;22(5):770. doi: 10.3390/ijerph22050770 (PMC12111452; doi:10.3390/ijerph22050770)
Supplement: Supplementary file 1 [file ijerph-22-00770-s001.zip › ijerph-3515542-supplementary.pdf]

Review

Supplementary file

Multimorbidity Management: A scoping review of interventions and health outcomes

Kagiso P Seakamela [1,2,*], Reneilwe G Mashaba [2], Cairo B Ntimana [1,2], Chodziwadziwa W Kabudula [3] and Tholene Sodi [4]

[1] Department of Pathology and Medical Sciences, Faculty of Health Sciences, School of Medicine, University of Limpopo, Polokwane, South Africa
[2] DIMAMO Population Health Research Centre, University of Limpopo, Sovenga St, Polokwane 0727, South Africa
[3] MRC/Wits Rural Public Health and Health Transitions Research Unit (Agincourt), Faculty of Health Sciences, School of Public Health, University of the Witwatersrand, Johannesburg, South Africa
[4] SAMRC-DSI/NRF-UL SARChI Chair: Mental Health and Society, University of Limpopo, Private Bag X1106, Polokwane, 0727, SOVENGA, South Africa
* Correspondence: seakamelakagiso@gmail.com

Table S1: Summary of included studies.

Author, year and Location	Types of study	Participants characteristics	Sample size	Female (N)	Interventions
**Adam *et al.*, 2019 [20] Switzerland, Netherlands, Belgium, Ireland**	Cluster Randomized control trial	Older adults (≥70years)	80	NR	Reduce Inappropriate Prescribing through, systematic medication and a software-based tool to predict adverse medication effects, advising safe and appropriate therapy using established STOPP/START criteria. Monitoring clinically relevant interactions and dosing. Drug discussion and adaptation with the prescribing physician. Shared decision-making with the patient.
**Afshar *et al.*,2015 [21] 28 countries of the World Health Survey (2003)**	Multi-stage clustering design	Participants aged 18 years or above	125404	NR	Better coordination and support through informed policy and planning of health care systems. Increase activities and expand

					measures to reduce the modifiable risk factors that are driving multi-morbidity prevalence.
**Anderson *et al.*, 2021 [22] Finland**	Cross-sectional	Participants with multimorbidity aged 20 - 69	3864	2017	Target lifestyle-associated risk factors for multimorbidity smoking, overweight, obesity, and physical inactivity in health interventions.
**Awuviry-Newton *et al.*, 2023 (23) Ghana**	Cross-sectional	People aged 50+ years	4446	2620	Incorporate physical activity into the routine care or post discharge care to reduce functional dysfunction. Policy interventions should incorporate a built environment (sidewalks) that will facilitate walking, organize regular community activities or programs for elderly adults that involves physical activities. Public health interventions and financial assistance aimed to promote and sustain physical activity among older adults.
**Barker *et al.*, 2018 [24] Australia**	Randomized case-control	Participants with multimorbidity	16	11	Exercise program consisting of aerobic and resistance exercises. Multidisciplinary professionals and participants' educational interventions to enhance general self-management skills and multimorbidity management.
**Berntsen *et al.*, 2019 [25] Norway**	A propensity score-matched controlled trial	Based on hospital electronic health record data of 439 multi-morbid (2014-2016)	1218	0	PACT interventions aimed to improve the management of multimorbidity through multi-sectoral collaboration, community engagement, patient-centred care, preventive strategies, chronic disease management, and the use of technology. Enhancing health outcomes, quality of life,

					and patient satisfaction while reducing healthcare costs
**Bleijenberg *et al.*, 2013 [26] Netherlands**	Cross-sectional	General practitioners and Practice Nurses	53	42	A mixed-methods procedure contributes to a more in-depth understanding of the barriers and facilitators of a proactive structured care programme. This study has increased our knowledge regarding the needs and experiences of GPs and PNs in providing proactive and structured care to frail older people in primary care.
**Blum *et al.*, 2021 [27] Switzerland, Netherlands, Belgium, and Ireland**	Cluster randomized controlled trial	Adults aged 70 years or more with multimorbidity	2008	NR	Pharmacotherapy optimization interventions that reduce inappropriate prescribing and improve patient outcomes.
**Bowling *et al.*, 2020 [28] England**	Retrospective cohort study	Participants with multimorbidity with a mean age of 73.8	6591	3678	Sustaining BP control may be an effective approach to slow multimorbidity progression and reduce the population burden of multimorbidity
**Camacho *et al.*, 2018 [29] UK**	Cluster randomized trial	Participants with mental–physical multimorbidity	382	145	Collaborative care to improve both depression and physical functioning in people with multimorbidity.
**Carballeira *et al.*, 2021 [30] Spain**	Randomized controlled clinical trial	Multimorbid patients aged 80 years and above	24	15	Low-volume cycling training to improve body composition and functionality in older people with multimorbidity
**Contant *et al.*, 2019 [31] Canada**	Randomized case-control	Participants with multimorbidity aged 50 years and above	281	141	Change in lifestyle specifically. Participants engagement in self-management/patient education programs or engaging in life-fulfilling activities). Promote individuals' general emotional well-being. Self-monitoring of the condition(s), physical and/or emotional. Promote constructive attitudes and approaches. Knowledge-based

					skills and techniques that participants acquire or relearn to help them manage and cope with disease-related symptoms and health problems. Social integration and support. Health services navigation.
**Coventry *et al.*, 2015 [32] United Kingdom**	Clustered randomized control trial	Participants with multimorbidity	36	NR	Collaborative care that incorporates brief low-intensity psychological therapy delivered in partnership with practice nurses in primary care can reduce depression and improve self-management of chronic disease in people with mental and physical multimorbidity.
**Dzomba *et al.*, 2023 [33] South Africa**	Population-based cross-sectional study	Adults aged between 18 and 40 years	3800	1131	Adaptation of healthcare delivery to monitor comorbidities and NCDs in HIV Individuals. Early initiation of longer-term life-course care. Improve the effectiveness of weightless campaigns and help in tackling obesity
**Espeland *et al.*, 2020 [34] USA**	Randomized case-control	Participants with multimorbidity aged 45 - 79 years	5145	3063	The multidomain interventions targeted at reducing caloric intake (1200–1800 based on initial weight) and increasing physical activity (>175 minutes per week through activities similar in intensity to brisk walking) to induce weight loss to average ≥7% at Year 1 and maintain this throughout follow-up.
**Espeland *et al.*, 2021 [35] USA**	Randomized controlled clinical trial	Multimorbid patients with T2DM and frailty aged 40 and above	5150	3063	Intensive lifestyle intervention or diabetes support and education. Reducing caloric intake and increasing physical activity to induce weight loss to average >7% at year 1 and to maintain this over time. Cardiometabolic risk factors (lipids, HbA1c, blood pressure) monitoring.

**Fortin *et al*., 2021 [36] Canada**	Pragmatic mixed-methods randomized controlled trial	Multimorbid patients aged 18 to 80 years	284	152	Health care workers training. Patient-centred care for persons with multimorbidity, self-management support, inter-professional collaboration, and patient's motivational approach. Individualized care plan.
**Freisling *et al*., 2020 [37] Denmark, France, Germany, Greece, Italy, the Netherlands, Norway, Spain, Sweden, and the UK**	Prospective cohort study	Aged 43 to 58 years	291,778	186738	Healthy lifestyles like regular physical activity should be implemented to reduce the risk of multimorbidity.
**Gallo *et al*., 2016 [38] USA**	Cluster-randomized, controlled trial	Multi-morbid patients aged 60 years and above	1204	727	Employ depression care managers to implement and monitor treatment response, adherence, and side effects among multi-morbid, depression according to standard guidelines. Depression management should be integrated into the care of older adults with multi-morbidity.
**Garvey *et al*., 2015 [39] Ireland**	Pragmatic feasibility randomized controlled trial	Participants with multimorbidity aged 40 years and above	50	32	OPTIMAL is effective in improving a range of outcomes for individuals with multimorbidity and contributes towards the evidence base on the effectiveness of interventions for people with multimorbidity. Occupational therapy led self-management support programme (OPTIMAL) employing following elements: 1. Weekly group meetings for a six-week period held in local community health centres. Occupational Therapy focus. Peer support. Goal setting and prioritization based on patient preferences OT

					interventions to support patient self-management used in the groups include Self-management. Fatigue and energy management. Managing stress, and anxiety, and maintaining mental health and well-being. Keeping physically active, healthy eating, managing medications, effective communication strategies, and goal setting.
**Gausi *et al.*, 2021 [40] South Africa**	Observational retrospective cohort study	Patients living with HIV and comorbid NCD before and upon enrolment into integrated clubs (IC)	246	187	Intensified NCD-specific health-promoting interventions, upon enrolment into integrated care, particularly for patients with advanced HIV disease or a long NCD history, to sustain NCD control in the long term.
**Gillespie *et al.*, 2022 [41] Ireland**	Randomized controlled trial	Participants with multimorbidity	149	103	OPTIMAL intervention, which includes management of fatigue, stress, diet, physical activity, and medication adherence. Goal setting and action planning to facilitate long-term changes to health behaviours. Materials to support their engagement (e.g. exercise booklets, get active your way, healthy eating, mental health).
**Hien *et al.*, 2014 [42] Burkina Faso**	Cross-sectional study	Elderly people aged ≥ 60	389	207	Reorganization of care issues in health systems in sub-Saharan Africa. Interventions that target individual diseases may not be appropriate for patients with multiple diseases. There is a need for Research to develop innovative interventions to reduce the burden of multimorbidity in sub-Saharan Africa

**Hirst *et la.*, 2021 [43] United Kingdom**	Prospective cohort study	Multimorbidity primary care patients with decreased renal function aged 60 years and older	861	468	A patient-centred approach to treatment and care of multiple comorbidities is required in treating multimorbidity. There is a need for Regular blood pressure monitoring to guide treatment to optimal blood pressure targets with antihypertensive medication.
**Huibers et al., 2022 [44] Netherlands**	Cluster randomized controlled trial	Older people (≥ 70 years) with polypharmacy (≥ 5 chronic medications)	139	73	Structured medication review based on the software-supported Systematic Tool to Reduce Inappropriate Prescribing (STRIP). Structured History taking of Medication use (SHiM) and collection of patient data including medical conditions, laboratory data and clinical parameters. Digitalize screening of pharmacotherapy through Clinical Decision Support System (CDSS). START and STOPP signals generated by the CDSS based on the patient data and current pharmacotherapy. Discussion of individualized medication optimization with the patient and attending physician.
**Jäge *et al.*, 2017 [45] Germany**	Cluster-randomized controlled trial	Health practitioners	22	16	Medication reviews to increase expert knowledge and feasibility of instruments for systematic medication reviews. Information material for patients to increase self-management abilities and reduce language barriers and difficulties of comprehension.
**Jäge *et al.*, 2017 [46] Germany**	Cluster-randomized controlled trial	Health practitioners	22	16	Training and resources for general practitioners. Identifying potential barriers and solutions for the implementation of the recommendations. Educational material for patients: Posters encouraging patients to take their medication list and “info-tool” for

					patients on a tablet PC. Implementation action plans.
**Jungo *et al.*, 2023 [47] Switzerland**	Cluster randomized clinical trial.	Patients were ≥65 years of age with three or more chronic conditions and five or more long-term medications.	43	9	A structured six-step medication review using STRIPA, a web-based electronic clinical decision support system based on the STOPP/ START criteria version 2. Monitor potential overuse, underuse, and misuse of drugs. Use STRIPA to generate recommendations to prevent drug-drug interactions and inappropriate dosages. The one-time intervention consisted of six steps. (1) Data on medications, chronic conditions, laboratory values, and vital data imported to STRIPA. (2) General practitioners verified and adapted the recorded information. (3) General practitioners used the drag/ drop function to link medications and conditions. (4) General practitioners ran the medication review. (5) General practitioners decide which recommendations to move forward. (6) At the next appointment, general practitioners implemented shared decision-making with patients.
**Jungo *et al.*, 2023 [48] Switzerland**	Cluster randomized controlled trial	General practitioners aged 40 years and above	43	9	General practitioners use the intervention at the individual patient level. Structured six-step medication review using STRIPA, a web-based electronic clinical decision support system based on the STOPP/ START criteria. Method for detecting potential overuse, underuse, and misuse of drugs, STRIPA generated recommendations to

					prevent drug-drug interactions and inappropriate dosages.
**Kamkuemah** ***et al.*****, 2022 [49]**<br>**South Africa**	Cross-sectional study	Adolescents and youth receiving treatment for HIV in primary care aged 15 - 24 years	92	70	Multi-sectoral interventions are required beyond the healthcare sector to reduce the impact of NCDs on health systems and broader societal development. Further studies are needed to assess risk factors at a broader socioecological level and explore multilevel determinants of HIV/ NCD comorbidity in adolescents and youth.
**Keetile** ***et al.*****, 2020 [50]**<br>**Botswana**	Cross-sectional	Participants aged 15 years and older	1178	813	Strengthen interventions encouraging healthy lifestyles such as non-consumption of alcohol, physical activity and healthy diets. A holistic approach of health care services to meet the needs of those suffering from multimorbidity.
**Khunti** ***et al.*****, 2021 [51]**<br>**United kingdom**	Randomized case-control	Participants with multimorbidity aged 40 years and older	353	NR	Movement through Active Personalized Engagement (MAP) program. Addressed key non-disease-specific self-management challenges and themes (mastering emotions, managing treatments, communication within health care). Support a long-term change in health behaviour, regular reminders and motivational text messages to the participants.
**Köberlein-Neu** ***et al.*****, 2016 [52]**<br>**Germany**	Cluster-randomized trial	Multimorbid patients aged 70 years and older	142	76	Inter-professional collaboration to increase medication safety. Working across disciplinary boundaries to allow for a decrease in drug-related problems and bring up aspects outside the purview of the primary care physician.

**Lanzeta *et al.*, 2016 [53] Spain**	Cluster randomized clinical trial	Patients with multimorbidity aged 45 years and older	140	45	Implementation of an integrated health care model for multimorbid patients based on improving communication between primary care and hospital professionals. Enhance continuity of care after hospitalization in coordination with primary care to avoid re-hospitalizations. Provide health education to improve self-management of each specific disease.
**Lea *et al.*, 2020 [54] Norway**	A randomized controlled trial	Multimorbid patients aged 70 years and older	386	208	Medicines reconciliations and reviews conducted by clinical pharmacists and multidisciplinary health teams to minimize symptoms of drug-to-drug interactions. Considering patients' characteristics and entire patient's drug list.
**Lear *et al.*, 2021 [55] Canada**	Single-blinded randomized clinical trial	Patients with multiple chronic diseases aged 60 and above	229	88	Internet-based self-management program using telephone nursing supports and integration within primary care
**Lin *et al.*, 2014 [56] USA**	Randomized controlled trial of a multimorbidity	Participants with depression and uncontrolled diabetes with multimorbidity	214	111	Self-management support, monitoring of disease indicators, and pharmacotherapy with frequent treatment adjustments to control depression, hyperglycaemia, hypertension, and hyperlipidaemia. Collaborative care for depression, chronic care model, and treat-to-target strategies initially developed for diabetes. Proactively monitoring clinical progress and use motivational and problem-solving approaches to support medication adherence, healthy eating, and physical activity. An electronic registry supported tracking of PHQ-9 scores and A1C, LDL, and

					BP levels, and flag patients who are not making good progress. Treatment protocols employing commonly used medicines guided consultant recommendations, and medication changes tailored to patient history and clinical response. Relapse prevention and maintenance plan.
**Lo *et al.*, 2020 [57] Taiwan**	Randomized controlled trial	Multimorbid patients aged 40 years and above	53	22	Individual face-to-face counselling session and educational brochure about the importance of regular exercise, the benefits of aerobic exercise training for multimorbidity, and physical activity recommendations/general aerobic exercise prescriptions based on WHO/ACSM guidelines
**Mackinnon *et al.*, 2023 [58] Ghana, Senegal, Mali, Cameroon , Niger, Gambia, Nigeria, Benin, South Africa, Spain and Egypt**	Cross-sectional	Multimorbid participants	436	173	Early detection strategies and tailored public health interventions that aim to reduce the disease burden.
**Majert *et al.*, 2024 [59] England**	Decentralized, two-armed, parallel-group, open-label randomized controlled pilot trial	Adults aged 70 years or more with multimorbidity	230	118	The decentralized trial design effectively engages older individuals with multiple health conditions and medication regimens, allowing them to participate in research from the comfort of their homes. The intervention successfully achieved a significant decrease in systolic blood pressure without adversely affecting participants' quality of life or cognitive function. These findings provide some reassurance about intensive blood pressure

					lowering in this under-represented patient group and could help inform the design of future studies.
**Matima** ***et al.*****, 2018 South Africa [60]**	Qualitative study	Patients with both HIV and T2D between 35 and 65 years	10	NR	Integration of chronic services and addressing social determinants of health may be the first steps towards alleviating patient burden and improving their access and utilization of these services. Further studies are necessary to explore multimorbidity beyond the context of HIV/T2D. Improved continuity of care and self-management of multi-morbidities included integration of chronic services, consolidated guidelines for healthcare workers, educational materials for patients, improved information systems and income for patients
**Mazya** ***et al.*****, 2019 [61] Sweden**	Randomized controlled trial	Elderly people with multimorbidity and high health care utilization and frailty	375	189	Interventions that counter frailty, such as physiotherapy, advice on diet, nutritional support, and pharmacological optimization in older patients with multimorbidity. Future research investigating personal factors known to influence health, such as self-efficacy, coping, and resilience in managing multimorbidity.
**Mbokazi** ***et al.*****, 2023 [62] South Africa**	Qualitative study	30 patients with HIV/NCD multimorbidity, 16 in Gugulethu (9 women, 7 men) and 14 in Bulungula (12 women, 2 men)	44	21	Decolonize individual illness behaviour theory developed in HICs and move towards acknowledging culturally embedded patterns of support and collaboration by incorporating Ubuntu, imbeko and other African social support theories into existing treatment burden

					models. Future interventions need to recognize the invaluable role that Ubuntu plays for people living with multimorbidity in South Africa and consider how existing social networks can be strengthened to enhance self-management capacity, especially in a changing health care landscape where patients are expected to take more responsibility for their own health
**McAiney *et al.*, 2022 [63] Canada**	Qualitative study	Older adults with multimorbidity and depressive symptoms	47	NR	Intervention implementation was facilitated by: (a) engaging the community to gain buy-in and adapt Community Assets Supporting Transitions to the local community contest. (b) planning, training, and research meetings. (c) facilitating engagement, building relationships, and collaborating with local partners. (d) ensuring availability of support and resources for Care Transition Coordinators. (e) tailoring of the intervention to individual client (i.e., older adult) needs and preferences. Implementation barriers included: (a) difficulties recruiting and retaining intervention staff; (b) difficulties engaging older adults in the intervention; (c) balancing tailoring the intervention with delivering the core intervention components; and (c) Care Transition Coordinators' challenges in engaging providers within clients' circles of care.
**McCarthy *et al.*, 2023 [64] Ireland**	Cluster randomized controlled trial	Participants aged 65 years and older.	404	237	Explore patients' perspectives and experiences of frequent medication

					switching and the impact this has on adherence and using them safely. Restricting access to certain medicines is an effective approach to tackling sub-optimal prescribing.
**McCarthy *et al.*, 2022 [65] Ireland**	Cluster randomized controlled trial	Participants aged 65 years and older with multimorbidity.	404	237	Primary care-based medication review intervention aims to reduce significant polypharmacy leading to the de-prescription of unnecessary medicines.
**McCarthy *et al.*, 2022 [66] Ireland**	Cluster randomized controlled trial	Participants aged 65 years and older with complex multimorbidity.	404	231	Support in identifying potentially inappropriate prescriptions. Provide treatment alternatives for identified inappropriate prescriptions, perform a brown bag medication review and record any problems identified and, assess and record patients' priorities for treatment
**Mercer *et al.*, 2016 [67] United Kingdom**	Phase 2 cluster RCT	Multimorbid patients aged 30–65 years	126	85	A whole-system intervention to improve the quality of life of primary care patients with multimorbidity in areas of high socioeconomic deprivation. Enhancing primary care through a whole-system approach may be a cost-effective way to protect quality of life for multimorbid patients in deprived areas.
**Miklavcic *et al.*, 2020 [68] Canada**	Pragmatic randomized controlled trial	Adults with multimorbidity aged 75 years or older	132	72	Six monthly group sessions at the community site, monthly intervention team case conferences and linked the client to other relevant health or social services.
**Mohamed *et al.*, 2021 [69] Kenya**	Cross sectional study	2003 healthy adults between 40 and 60 years of age	2003	1082	Concerted efforts to develop strategies for the planning, prevention and management of modifiable risk factors that drive the high

					prevalence of multimorbidity. Designing integrated chronic care models. Further research.
**Mpinganjira *et al.*, 2023 [70]**<br>**South Africa**	Longitudinal Study	Individuals aged ≥ 40 years	5059	2712	There is an urgent need to integrate alcohol interventions in the management of NCDs and multimorbidity and such interventions should include an objective assessment of alcohol consumption
**Nagl *et al.*, 2012 [71]**<br>**Germany**	Cross-sectional study	Multimorbid patients aged 72 or above	1937	1033	Considering pharmaceuticals and outpatient physician services cost when making future-oriented decisions on resource allocation utilization in the elderly with multimorbidity.
**Nkoka *el at.*, 2024 [72]**<br>**Malawi**	Cross-sectional study	Individuals aged 15 years and above	9849	5562	Design and deliver effective, holistic health care for people living with multiple physical and mental health conditions in rural and urban Malawi.
**Odland *et al.*, 2020 [73]**<br>**Burkina Faso**	Cross-sectional study	Adults ≥40 years	2604	1304	Investment by researchers, development agencies and national governments to priorities understanding of multimorbidity, prevention in lower income settings.
**Odland *et al.*, 2022 [74]**<br>**Iran**	Longitudinal cohort study	Participants aged 40–75 years.	47 883	27416	Politicians and policymakers in low-income countries, and with global health funding need to focus on chronic conditions and multimorbidity in the years to come to avoid the massive burden multimorbidity is likely to place on health systems.
**Oh *et al.*, 2024 [75]**<br>**USA**	Cross-sectional	Multimorbid patients aged (≥55years)	400	211	Interventions aimed at minimizing distress, which has been associated with a lower adherence to treatment, leading to worsening of disease severity and outcomes.

					Raising awareness on distress and related mental health problems and promoting utilization of mental health care among people with multimorbidity.
**Olanrewaju *et al.*, 2022 [76]**<br>**China, Ghana, India, Mexico, Russia and South Africa.**	Cross-sectional study	Adults aged≥50 years	34129	17065	Future intervention studies with long follow-up periods are warranted to examine whether addressing the identified mediators can improve QoL in older people with multimorbidity in LMICs.
**Oni *et al.*, 2015 [77]**<br>**South Africa**	Cross-sectional study	Participants aged 18 years and above with multimorbidity	14 364	10198	The current models of health care delivery need to be re-examined, and patient-centred models of integration evaluated including bidirectional screening of commonly co-morbid conditions in routine clinical practice. Furthermore, research into possible causal underlying mechanisms where unknown; and the implications for diagnosis and treatment, adherence, health outcomes, and capacity for behaviour change is required.
**Ose *et al.*, 2019 [78]**<br>**Germany**	18-month, multi-centre, two-armed, open-label, patient-randomized parallel-group superiority trial	Multimorbid patients aged 68 years and older with diabetes.	495	228	Care management program as add on to normal treatment aimed at improving diabetes self-care behaviour among patients with type 2diabetes and multiple comorbidities. Multifaceted intervention involving focus groups with PCP physicians and specialist care providers, and the active engagement of representatives of local patient self-help groups in formulating assessment contents and identifying community resource. Ensure continuity of care and the collaboration between involved

					health professionals. Facilitates shared learning and coordinated standardization of healthcare delivery across practices. Support patients via home visits and telephone-monitoring to help them locate additional services within the health system or the broader community according to patient's individual preferences and needs
**Otieno *et al.*, 2023 [79] Ghana and South Africa**	Retrospective cross-sectional study	Participants aged 50 years and older	4190	2263	Primary, secondary, and tertiary prevention of functional disability at the population and individual level should target older persons with or at risk of concordant and discordant cardiometabolic multimorbidity comprising hypertension, abdominal obesity, diabetes, cataracts, arthritis and multimorbidity of angina, chronic lung disease, asthma, and depression
**Pati *et al.*, 2017 [80] India**	Cross-sectional study	Patients attending 40 primary healthcare	1649	729	National NCD program should design customized or tailor-made disease or case management protocols for patients with multiple morbidities, as most of the patients might be consulting individual providers thus resulting in fragmented care and suboptimal outcomes. Specific treatment guidelines or patient education programs for persons with high-frequency clusters or with clusters of diseases are particularly difficult to be treated. Evidence-based research on better care models for patients with multimorbidity to reduce the potential for drug-drug and drug-disease interactions should be initiated

**Petersen *et al.*, 2019 [81] South Africa**	Cross-sectional study	Patients aged 18 years or older;	2549	1950	Integrate mental health screening and treatment into HIV-care platforms, particularly in resource-deprived settings, should be a public health priority
**Portz *et al.*, 2017 [81] USA**	Retrospective study	Adults from palliative care research cooperation	381	171	Improved measures to include both diagnosis and illness severity for capturing multimorbidity and effective approaches to address symptom burden in older adults with life-limiting illness and multimorbidity are needed
**Prenissl *et al.*, 2022 [83] India**	Representative cross-sectional study	Individuals aged 15 to 49 years	712822	617374	Reforms to shift the health system's focus away from episodic care for acute conditions towards longitudinal, integrated, and person-centred care.
**Read *et al.*, 2021 [84] Austraila**	Randomized trial	Participants aged 65 years	302	NR	Evidence is emerging that iCBT (internet-delivered cognitive-behaviour therapy) can be implemented successfully in routine clinical care, as part of a stepped-care approach. Remote interventions are needed for long-term results and to provide a clinical and cost-effective way to prevent depression in the context of multimorbidity.
**Read *et al.*, 2020 [85] Australia and New Zealand**	Randomized control trial	Participants aged 65 with 2 or more conditions chronic physical conditions	302	212	iCBT to prevent depressive disorder. Specifically, iCBT was effective in preventing cases of depressive disorder during the first six months despite low rates of depressive disorders in the sample. iCBT can prevent or at least delay depressive disorder in older adults with multimorbidity. Further investigations are required to understand whether there are longer-term benefits of the

					intervention and whether the intervention is cost-effective
**Riegel *et al.*, 2016 [86] USA**	Randomized control trial	Hospitalized heart failure patients with multimorbidity aged 18 and above	100	37	Transitional to decrease readmissions for this population. Accountable Care Organizations to provide cost-effective, clinic-based services for general multimorbid populations. Interdisciplinary primary care and interdisciplinary teams for nursing home residents. Patient-centred medical homes and Guided Care.
**Roche *et al.*, 2017 [87] South Africa**	A descriptive cross-sectional study	Patients admitted to the internal medicine department, with a mean age of 49 years	491	282	Improve the health of communities, through partnerships between doctors, community health providers and patients with their families. Educational interventions understanding of multimorbidity, including the patient perspective, in medical education and health system reform.
**Romano *et al.*, 2021 [88] low-and middle income countries**	Cross-sectional study	Adults aged ≥18 years	20,198	10927	Reduce obesity among older adults as it is associated with increased odds for multimorbidity. Future longitudinal research is required to assess the impact of dietary reduction on multimorbidity incidence
**Roomaney *et al.*, 2022 [89] South Africa**	Cross-sectional study	Individuals over the age of 15 years	27896	16422	The gains in improving adherence to antiretroviral should be expanded to include compliance with lifestyle/behavioural modifications to blood pressure and glucose control, as well as adherence to anti-hypertension and anti-diabetic medication. Improve the early diagnosis and treatment of disease in the South African population

**Roomaney *et al.*, 2022 [90] South Africa**	Cross-sectional study	10 336 people who participated in the Adult Health Questionnaire	10336	6126	Reducing the high prevalence of single diseases such as hypertension and (simultaneously targeting people with existing diseases to reduce their chances of becoming multimorbid. More studies are needed to identify common disease clusters and multimorbidity trends to assist in the endeavour of targeting high-risk people. Information is needed on how emerging diseases such as COVID-19 may affect people with multimorbidity in South Africa.
**Roomaney *et al.*, 2023 [91] South Africa**	Cross-sectional study	Participants aged 15 years and older who had more than one disease condition	2368	1694	Integrated care is needed, evidenced by the largest disease class being an overlap of chronic infectious diseases and non-communicable diseases. There's a need to address hypertension by tackling the risk factors associated with hypertension could avert an epidemic of multimorbidity.
**Roomaney *et al.*, 2022 [92] South Africa**	Cross-sectional study	People aged 15 years and older (N = 27,042)	27042	15333	Integrated models of care are needed to prevent, manage and optimize the treatment of NCD multimorbidity.
**Salari *et al.*, 2022 [93] Switzerland, Belgium, Ireland and the Netherland.**	cluster-randomized trial	Elderly people (≥70years)	1918	857	Reduce potential inappropriate prescribing and DRAs, using a software-based pharmacotherapy optimization intervention based on the STOPP/START criteria. Optimization of pharmacotherapy as a means of reducing hospital admissions and improving other patient-relevant outcomes.
**Salisbury *et al.*, 2018 [17] England and Scortland**	Pragmatic cluster-randomized trial	Elderly people (≥70years)	1546	773	Creating guidelines, which take into account multimorbidity as disease-specific guidelines, can be inappropriate for patients with coexisting conditions. If each condition

					is considered in isolation, patients can be prescribed numerous drugs and lifestyle changes and are expected to attend frequent healthcare appointments. Therefore, treatment itself can represent an excessive burden for patients with multimorbidity, alongside their burden of illness. Furthermore, segmentation of care by disease means that health care for these patients is often fragmented and poorly coordinated. Establishing patient-centred health care for patients with multi-morbidity to improve health and wellbeing.
**Sallevelt *et al.*, 2022 [94] Switzerland, Belgium, Ireland and the Netherlands**	Cluster-randomized controlled trial	Patients (>76 years) with polypharmacy and multimorbidity	963	482	Future improvements in the shared decision-making process between the patient, pharmacist and physician. Implementation of STOPP/ START recommendations. Improvements in medication reconciliation across health care settings to avoid unintentional re-prescription of medication.
**Sallevelt *et al.*, 2021[94] Switzerland, Belgium, Ireland, the Netherlands**	Cluster-randomized controlled trial	Participants aged ≥ 70 years, multimorbidity	821	197	The involvement of an expert team in translating population-based Screening Tools of Older Persons' Prescription signals to individual patients is essential, as more than half of the signals for potential overuse, underuse, and misuse were not deemed clinically appropriate in a hospital setting.
**Salm *et al.*, 2023 [95] Germany**	Framework analysis	Patients aged 77 years and above with multimorbidity	6	2	Frequent meetings in which healthcare professionals come together to discuss and collaborate on the diagnosis, treatment, and management of a particular patient's case (case conference) to clearly defined

					responsible party, to improve coordination of care. Improve and advance coordination of ambulatory care at the patient level to avoid redundant inpatient stays, overuse of outpatient services, the underuse of vaccinations and potential prescribing omissions. Patient are supported through self-management strategies, with close guidance from their caregivers.
**Singh *et al.*, 2023 [96] South Africa**	Cross-sectional study	Participants aged 15 and above within the survey area	18041	12229	In South Africa, the effective health systems that have catered well to individuals with HIV should be leveraged to tackle the unaddressed requirements of those with hypertension and diabetes. Merge primary healthcare services and devise innovative, cost-effective strategies for diagnosing and treating multiple ailments simultaneously
**Smith *et al.*, 2022 [97] low-and middle income countries**	Cross-sectional study	Adults aged 50 years and above	34129	17781	Future longitudinal and intervention research is to evaluate the temporal relationships and the impact of addressing the potential mediators of depression among older individuals with multimorbidity in Low- and Middle-Income Countries (LMICs). Raising awareness about the significance of integrating physical activity engagement among healthcare professionals in Low- and Middle-Income Countries (LMICs) is essential.
**Stanton *et al.*, 2024 [98] South Africa**	A population-based study	Adolescents and adults, aged 15 years and older	14008	9573	Integrating prevention measures for Non-Communicable Diseases (NCDs) and addressing multiple health conditions within healthcare strategies can potentially

					enhance HRQoL. Healthcare providers should be equipped to manage multimorbidity by prioritizing the quality of life while addressing the challenges of managing multiple concurrent conditions. Prevention initiatives tailored to target diseases and health conditions that have the most detrimental effects on Health-Related Quality of Life (HRQoL). Allocating resources to initiatives aimed at preventing Non-Communicable Diseases (NCDs) to enhance Health-Related Quality of Life (HRQoL) in South Africa and similar settings where there is an intersection of NCD and infectious disease epidemics.
**Stubbs *et al.*, 2018 [99]**	Population-based cross-sectional study	Amongst participants aged 50 and above	34129	17781	Need for affordable, population-wide integrated interventions to manage stress among individuals with chronic conditions. Healthcare systems to adjust to the growing challenge of multimorbidity and deteriorating mental health. An essential initial action involves increasing awareness among primary and mental healthcare providers in LMICs about the significance of acknowledging chronic conditions and perceived stress. Affordable, broad-scale interventions, such as promoting healthy lifestyles, to preventing and managing chronic conditions, and subsequently, multimorbidity. Regular physical activity, may also serve to reduce stress levels.

**Sum *et al.*, 2019 [100] middle income countries**	Cross-sectional analysis	Young adults (18-49), those aged 50-64, and elderly (aged 65 and above)	47443	23877	Health policies to improve the accessibility of resources and health care funding to individuals with NCDs with the largest effects on increasing outpatient visits and hospitalizations and lowering QoL. Develop integrated care delivery models tailored for common chronic conditions. Research to track the evolution of multimorbidity patterns across the lifespan, in tandem with economic, demographic, and epidemiological shifts, employing prospective methodologies to offer insights into causation and avenues for preventing the onset or progression of certain conditions.
**Takahashi *et at.*, 2016 [101] USA**	Randomized controlled trial	Adults with multimorbidity aged 63 years	130	94	Motivate for use of pedometers and physical goal setting to lose weight as an intervention
**Tanke *et al.*, 2019 [102] seven high-income OECD member countries (cross country)**	Cross-sectional Descriptive study	High-cost patients (all patients with multimorbidity)	NR	NR	Better coordination of care before, during and after an inpatient admission. More research focusing on identifying homogenous patient groups within the top 5% highest spenders and studying their care pathways may yield many opportunities to identify promising ways for improvements. Cross-country comparisons also can help because they create larger populations solving possible small-n diseases.
**Tayu *et al.*, 2015 [103] Ghana, China, India, Mexico, Russia, South Africa**	Cross-sectional	Participants aged 18 years and older	39213	39213	Further research is required to understand the additional demands of multimorbidity on health systems and the cost-effectiveness of different strategies to reduce the burden of multimorbidity on individuals and health

					systems in LMICs. Universal health coverage in LMICs for the elderly who are more likely to have multiple NCDs.
**Teljeur *et al.*, 2013 [104]**	Cluster randomized controlled trial	Patients T2DM with multimorbidity aged >50 years	424	227	Clinicians and educators need to support patients in prioritizing management strategies. Diabetes should not be treated in isolation. Multimorbidity needs to be considered when delivering patient education, giving risk-reduction advice and making treatment recommendations.
**Thorn *et at.*, 2020 [105] Scotland and England**	Pragmatical 3D cluster randomized trial	Multimorbid patients aged (≥70years)	1546	773	Implementing cost-effectiveness of a patient-centred approach to managing multimorbidity in primary care.
**Tomita *et al.*, 2021 [106] Tanzania**	Population-based study	Adults aged 40 and above	2299	1555	As public health resources remain scarce, reducing costly inpatient hospitalization requires multilevel interventions that address clinical- and structural-level challenges (e.g. food insecurity) to mitigate multimorbidity and promote long-term healthy independent living among older adults in Tanzania. Restructure the social welfare framework and establish culturally sensitive healthcare systems capable of managing multimorbidity amidst the unprecedented urbanization occurring in Tanzania.
**Verdoorn *et al.*, 2019 [107] Netherlands**	Randomized case-control	Participants with multimorbidity	629	238	Clinical medication review focused on personal goals, quality of life, and health problems
**Wang *et al.*, 2019 [108] South Africa**	Longitudinal study	Women and men aged 40 and above	5,890	3080	Sociodemographic factors play a significant role in the health and quality of life outcomes

					among elderly South Africans, highlighting the need for increased efforts to address health-limiting conditions like difficulties with ADLs and chronic multimorbidity. Geriatric health promotion programs focus on biopsychosocial factors to enhance health and quality of life among elderly populations.
**Waterhouse *et al.*, 2017 [109]**<br>**South Africa**	Retrospective study	Adults aged 50 years and older	3842	1845	Understanding the connections between socioeconomic status, illness diagnosis, and disability will help the South African health system provide adequate care for its older population. Health interventions should specifically target those with multiple non-communicable diseases (NCDs) through preventative, rehabilitative, and palliative care to minimize and manage disability. Additionally, to better allocate scarce resources, more research is needed to explore the sequence of NCD diagnoses and the timing of related disabilities.
**Webel *et al.*, 2019 [110]**<br>**USA**	Randomized Clinical Trial	PLWH and other chronic conditions aged 50 years and above	179	95	Strategies aimed at reducing symptom distress and improving aspects of coping and self-management behaviours, decreased social isolation, increased ability to self-manage illness or increase in completion of advance care planning. Inclusion of an expert workforce, teamwork among primary care clinicians and specialists, adherence to evidence-based guidelines, addressing financial barriers, and congruence with the prevailing healthcare culture.

**Weir** ***et al.*****, 2024 [111] Switzerland**	Randomized Clinical Trial	Older adults (≥65 years of age), with multimorbidity)	298	139	Encouraging adults' adherence to medications and de-prescribe.
**Widmann** ***et al.*****, 2017 [112] Somalia**	Randomized controlled intervention trial	Comorbid psychopathology	330	NR	Assessment of comorbid psychopathology and integrated treatments for substance use and comorbid mental health problem
**Wittink** ***et al.*****, 2016 [113] USA**	Randomized control study	Patients aged 40 and older, with multimorbidity	60	44	Technology interventions at the point-of-care, to educate patients and gather their preferences, empower patients to discuss aspects of their health, such as daily challenges. With the help of technology, patients and primary care providers (PCPs) could communicate more effectively about what matters most to patients, focusing earlier in the visit on practical aspects of managing chronic disease. Ideally, these conversations could help tailor treatments and resources to the patient's specific circumstances, leading to better outcomes.
**Wong** ***et al.*****, 2021 [114] South Africa**	Cross-sectional, population-based multimorbidity study	The participants, who were 15 years or older,	17118	11618	Targeted public health programs and scalable biomedical innovations are crucial to reducing the burden of specific diseases and multimorbidity for better population health. Investing in population science to understand the interactions between infectious and non-communicable diseases and to use this knowledge to create effective biomedical and public health strategies to enhance the health of African populations
**Yao** ***et al.*****, 2021[115] China**	Prospective cluster randomized trial	Patients with multimorbidity aged >70 years	1890	718	Implementing mHealth technology-based integrated care approach that facilitates to reduced meaningful clinical adverse events

					in older patients with AF and multimorbidity.
**Zechmann *et al.*, 2020 [116]**<br>**Switzerland**	Cluster-randomized clinical study	Patients with multimorbidity aged >76 years	334	150	Straightforward and patient-centred de-prescribing procedure.
**Zgibor *et al.*, 2017 [117]**<br>**USA**	Non-blinded cluster randomized trial	Patients with multimorbidity aged >70 years	462	407	Because older adults are at risk for multiple chronic disabling conditions, programs must consider the problem of multimorbidity. Community health workers training to deliver prevention interventions to older adults in community settings. Greater efforts to reach individuals in need of intervention with greater emphasis on goal setting and follow-up.

NR~ Not Reported, NCDs~ Non communicable diseases, GP~ General Practitioner, PN~ Professional Nurse

Table S2. The number of participants by study type

Study type	Number of participants
Cross-sectional	1037586
Randomized case-control	37002
Cohort(s)	344359
Longitudinal	10949
Qualitative	101
Population-based	16307

Table S3. PubMed search strategy

**Query**	**Results**
multimorbidity<br>"multimorbid"[All Fields] OR "multimorbidities"[All Fields] OR "multimorbidity"[MeSH Terms] OR "multimorbidity"[All Fields]	12,452
multiple conditions<br>("multiple"[All Fields] OR "multiples"[All Fields]) AND ("condition"[All Fields] OR "condition s"[All Fields] OR "conditions"[All Fields])	173,306
"intervention s"[All Fields] OR "interventions"[All Fields] OR "interventive"[All Fields] OR "methods"[MeSH Terms] OR "methods"[All Fields] OR "intervention"[All Fields] OR "interventional"[All Fields]	10,945,454
("multimorbid"[All Fields] OR "multimorbidities"[All Fields] OR "multimorbidity"[MeSH Terms] OR "multimorbidity"[All Fields] OR (("multiple"[All Fields] OR "multiples"[All Fields]) AND ("condition"[All Fields] OR "condition s"[All Fields] OR "conditions"[All Fields]))) AND ("intervention s"[All Fields] OR "interventions"[All Fields] OR "interventive"[All Fields] OR "methods"[MeSH Terms] OR "methods"[All Fields] OR "intervention"[All Fields] OR "interventional"[All Fields])	76,746
((multimorbidity) OR (multiple conditions)) AND (interventions) Filters: Free full text, Full text, Clinical Trial, Randomized Controlled Trial, Humans, English<br>(("multimorbid"[All Fields] OR "multimorbidities"[All Fields] OR "multimorbidity"[MeSH Terms] OR "multimorbidity"[All Fields] OR (("multiple"[All Fields] OR "multiples"[All Fields]) AND ("condition"[All Fields] OR "condition s"[All Fields] OR "conditions"[All Fields]))) AND ("intervention s"[All Fields] OR "interventions"[All Fields] OR "interventive"[All Fields] OR "methods"[MeSH Terms] OR "methods"[All Fields] OR "intervention"[All Fields]	1,623

OR "interventional"[All Fields])) AND ((ffrft[Filter]) AND (clinicaltrial[Filter] OR randomizedcontrolledtrial[Filter]) AND (fft[Filter]) AND (humans[Filter]) AND (english[Filter]))	

Table S4. Preferred Reporting Items for Systematic reviews and Meta-Analyses extension for Scoping Reviews (PRISMA-ScR) Checklist

SECTION	ITEM	PRISMA-ScR CHECKLIST ITEM	REPORTED ON PAGE #
**TITLE**			
Title	1	Identify the report as a scoping review.	1
**ABSTRACT**			
Structured summary	2	Provide a structured summary that includes (as applicable): background, objectives, eligibility criteria, sources of evidence, charting methods, results, and conclusions that relate to the review questions and objectives.	1
**INTRODUCTION**			
Rationale	3	Describe the rationale for the review in the context of what is already known. Explain why the review questions/objectives lend themselves to a scoping review approach.	2
Objectives	4	Provide an explicit statement of the questions and objectives being addressed with reference to their key elements (e.g., population or participants, concepts, and context) or other relevant key elements used to conceptualize the review questions and/or objectives.	2
**METHODS**			
Protocol and registration	5	Indicate whether a review protocol exists; state if and where it can be accessed (e.g., a Web address); and if available, provide registration information, including the registration number.	2
Eligibility criteria	6	Specify characteristics of the sources of evidence used as eligibility criteria (e.g., years considered, language, and publication status), and provide a rationale.	3
Information sources*	7	Describe all information sources in the search (e.g., databases with dates of coverage and contact with authors to identify additional sources), as well as the date the most recent search was executed.	2-3
Search	8	Present the full electronic search strategy for at least 1 database, including any limits used, such that it could be repeated.	Supplement pages 29-30
Selection of sources of evidence†	9	State the process for selecting sources of evidence (i.e., screening and eligibility) included in the scoping review.	4

SECTION	ITEM	PRISMA-ScR CHECKLIST ITEM	REPORTED ON PAGE #
Data charting process‡	10	Describe the methods of charting data from the included sources of evidence (e.g., calibrated forms or forms that have been tested by the team before their use, and whether data charting was done independently or in duplicate) and any processes for obtaining and confirming data from investigators.	3
Data items	11	List and define all variables for which data were sought and any assumptions and simplifications made.	3
Critical appraisal of individual sources of evidence§	12	If done, provide a rationale for conducting a critical appraisal of included sources of evidence; describe the methods used and how this information was used in any data synthesis (if appropriate).	N/A
Synthesis of results	13	Describe the methods of handling and summarizing the data that were charted.	3
**RESULTS**			
Selection of sources of evidence	14	Give numbers of sources of evidence screened, assessed for eligibility, and included in the review, with reasons for exclusions at each stage, ideally using a flow diagram.	3-4
Characteristics of sources of evidence	15	For each source of evidence, present characteristics for which data were charted and provide the citations.	Pages 5-6 and supplement pages 1-28
Critical appraisal within sources of evidence	16	If done, present data on critical appraisal of included sources of evidence (see item 12).	N/A
Results of individual sources of evidence	17	For each included source of evidence, present the relevant data that were charted that relate to the review questions and objectives.	Supplement pages 1-28
Synthesis of results	18	Summarize and/or present the charting results as they relate to the review questions and objectives.	7-12
**DISCUSSION**			
Summary of evidence	19	Summarize the main results (including an overview of concepts, themes, and types of evidence available), link to the review questions and objectives, and consider the relevance to key groups.	12-14
Limitations	20	Discuss the limitations of the scoping review process.	14
Conclusions	21	Provide a general interpretation of the results with respect to the review questions and objectives, as well as potential implications and/or next steps.	14
**FUNDING**			
Funding	22	Describe sources of funding for the included sources of evidence, as well as sources of funding	14

SECTION	ITEM	PRISMA-ScR CHECKLIST ITEM	REPORTED ON PAGE #
		for the scoping review. Describe the role of the funders of the scoping review.	

JBI = Joanna Briggs Institute; PRISMA-ScR = Preferred Reporting Items for Systematic reviews and Meta-Analyses extension for Scoping Reviews.

* Where *sources of evidence* (see second footnote) are compiled from, such as bibliographic databases, social media platforms, and Web sites.

† A more inclusive/heterogeneous term used to account for the different types of evidence or data sources (e.g., quantitative and/or qualitative research, expert opinion, and policy documents) that may be eligible in a scoping review as opposed to only studies. This is not to be confused with *information sources* (see first footnote).

‡ The frameworks by Arksey and O'Malley (6) and Levac and colleagues (7) and the JBI guidance (4, 5) refer to the process of data extraction in a scoping review as data charting.

§ The process of systematically examining research evidence to assess its validity, results, and relevance before using it to inform a decision. This term is used for items 12 and 19 instead of "risk of bias" (which is more applicable to systematic reviews of interventions) to include and acknowledge the various sources of evidence that may be used in a scoping review (e.g., quantitative and/or qualitative research, expert opinion, and policy document).
